# Risk of bone fracture by using dipeptidyl peptidase-4 inhibitors, glucagon-like peptide-1 receptor agonists, or sodium-glucose cotransporter-2 inhibitors in patients with type 2 diabetes mellitus: a network meta-analysis of population-based cohort studies

**DOI:** 10.3389/fendo.2024.1410883

**Published:** 2024-10-11

**Authors:** Mohamed E. A. Mostafa, Tariq Alrasheed

**Affiliations:** ^1^ Department of Anatomy, Faculty of Medicine, University of Tabuk, Tabuk, Saudi Arabia; ^2^ Department of Anatomy, Faculty of Medicine, Cairo University, Cairo, Egypt; ^3^ Department of Internal Medicine, Faculty of Medicine, University of Tabuk, Tabuk, Saudi Arabia

**Keywords:** T2DM, DPP-4 inhibitors, GLP-1 receptor agonists, SGLT-2 inhibitors, bone fracture

## Abstract

**Background:**

Type 2 diabetes mellitus (T2DM) is linked to a heightened likelihood of experiencing fractures. It is crucial to ascertain whether medications used to lower blood sugar levels can elevate the risk of fractures. We aimed to investigate and compare the effects of glucagon-like peptide 1 receptor agonists (GLP-1RA), Dipeptidyl Peptidase-4 Inhibitors (DPP-4i), and Sodium-Glucose Cotransporter-2 Inhibitors (SGLT-2i) on the fracture risk in patients with T2D in the real world.

**Methods:**

A network meta-analysis conducted an inclusive literature search in PubMed, Scopus, Web of Science, and Cochrane Library to select appropriate population-based cohort studies that investigated the risk of bone fractures of (GLP-1RA), (DPP-4i) or (SGLT-2i) in the real world. A network meta-analysis (NMA) was performed using R software to investigate the risk of total fractures as a primary outcome among patients who used (GLP-1RAs), (SGLT-2i) or (DPP-4i) versus each other or other glucose-lowering medications (GLMs). The odds ratio (OR) and 95% confidence interval (CI) were summarized overall network and for each pairwise direct and indirect comparison. The surface under the cumulative ranking curve (SUCRA) with the P-scores was calculated for each treatment in the network meta-analysis to detect their cumulative ranking probabilities in lowering the risk of total fractures.

**Results:**

In our NMA, we identified a set of 13 population-based cohort studies comprising a total of 1,064,952 patients. The risk of fracture was identified with the follow-up duration for each class. We found a significant decrease in the fracture risk by about 87% associated with patients who used SGLT2 inhibitors in combination with other glucose-lowering medications, followed by SGLT2 inhibitors alone by about 67%, then GLP-1 receptor agonists by about 60%, and at last DPP-4 inhibitors by about 55%.

**Conclusion:**

Our study’s collective findings suggest a significant association of the low risk of fracture with the use of SGLT2i with other GLMs combination, SGLT2i alone, GLP-1RA, and DPP-4i, respectively. This population-based analysis offers the best available evidence and might be helpful for clinicians in the decision of the most suitable T2DM treatment strategies, especially for elderly type 2 diabetic patients, as they may be safe in terms of fracture.

**Systematic review registration:**

https://www.crd.york.ac.uk/prospero/, identifier CRD42023448720.

## 1 Introduction

Type 2 diabetes among the elderly is acknowledged as a significant public health issue. The rising average lifespan and the persistent progression of type 2 diabetes are contributing factors to the growing occurrence of diabetes. Globally, approximately 135.6 million individuals aged 65 and above are estimated to have diabetes, with this figure projected to surge to 276.2 million by the year 2045 (1).

Both male and female patients diagnosed with T2DM have a heightened susceptibility to fractures when compared to individuals without diabetes (2). The occurrence of fractures in individuals with T2DM was found to be strongly linked to profound disability, increased societal impact, and a decline in quality of life (QoL) (3).

The increased fracture risk observed in individuals with T2DM could potentially be due to compromised bone quality or diminished bone strength, which can be influenced by the diverse impacts of GLMs on the metabolism of bone. It is of utmost importance to ascertain whether the use of GLMs can elevate fracture risks. Extensive research has been conducted in this area, and several studies have documented the impact of various GLMs on the risk of fractures in T2DM (4–7).

Denmark introduced glucagon-like peptide-1 receptor agonists (GLP-1 RA) and dipeptidyl peptidase 4 inhibitors (DPP-4i) in 2007, and since then, there has been a constant influx of novel medications belonging to these therapeutic classes (8). In recent times, GLP-1 RAs have arisen as a recommended therapy for individuals with T2DM and cardiovascular disease (9, 10). Additionally, they are utilized for weight loss purposes (11). Some cohort studies (12, 13) and meta-analyses (14, 15) have indicated that GLP-1 RAs have neutral effects on fracture risk. Nonetheless, one specific meta-analysis confirmed a decreased risk of fractures as a link to the use of GLP-1 RAs (16).

Sodium-glucose co-transporter-2 (SGLT2) inhibitors are the more recent class of drugs approved as blood glucose-lowering agents (10) and a recognized group of oral antidiabetic medications that effectively decrease the level of blood sugar by enhancing glucose elimination by urine. This is achieved by inhibiting the activity of SGLT2 in proximal tubules in the kidneys (17). SGLT2 inhibitors have garnered notable interest due to their remarkable ability to reduce cardiovascular disease (CVD) events alongside their glucose-lowering properties, all while posing a minimal risk of hypoglycemia (18). Considering these attributes, these medications could be deemed as viable therapeutic choices for elderly individuals diagnosed with type 2 diabetes. However, they have been proposed to increase fracture risk making their choice of conflicting choices (19).

Various authoritative organizations have endorsed dipeptidyl peptidase (DPP)-4 inhibitors as secondary treatment options for older individuals living with T2DM (20). Previous investigations concentrating on older adults with T2DM have demonstrated the effectiveness and safety of DPP-4 inhibitors, with minimal occurrences of hypoglycemic events and no elevated risk of bone fractures. Additionally, the use of DPP-4 inhibitors has been linked to a neutral risk of cardiovascular complications and mortality (21). Moreover, numerous studies have further reported no significant correlation between DPP-4 inhibitors and fracture risk (22–24). However, a subset of studies has even suggested a reduced risk of fractures among individuals using DPP-4 inhibitors compared to those not using them (25, 26).

Research endeavors aimed at examining the impact of diverse antidiabetic medications on fracture risk often encounter challenges related to confounding factors and inadequate durations of follow-up (27). Besides, The preceding meta-analyses have studied the effect of SGLT-2i, GLP-1 RAs, and DPP-4i on fracture risk among diabetic patients, but their results have displayed inconsistency (15, 28, 29). Also, given the recent COVID-19 pandemic and how the typical osteo-metabolic phenotype is characterized by a high rate of hypocalcemia, hypovitaminosis D and high prevalence of vertebral fractures, it is empirical to choose the most appropriate T2DM medication among patients with diabetes, who are known to have a worse prognosis from COVID-19, to prevent further fractures or bone damage (30).

Given these circumstances, the existing data are inadequate to justify the decision at present of the most suitable therapeutic strategies for elderly diabetics in routine clinical practice.

Thus, we conducted a network meta-analysis of real population-based cohort studies to investigate and compare the effects and relationship between SGLT-2i, GLP-1 RAs, and DPP-4i on bone fracture risk in patients with T2DM in the real world. Our objective was to investigate the bone fracture rate for each drug therapy and compare the collective bone fracture between those three classes. We aimed to present the odds ratio of total bone fractures associated with each treatment in comparison to other glucose-lowering medications to help clinicians make more evidence-based decisions while choosing T2DM therapy especially for high fracture risk patients.

## 2 Methods

The research protocol for this study was registered with the International Prospective Register of Systematic Reviews (PROSPERO) under registration number CRD42023448720.

The Preferred Reporting Items for Systematic Reviews and Network Meta-Analyses (PRISMA-NMA) checklist guidelines (31) were followed to ensure a systematic approach to the search process and reporting of the findings shown in **Supplementary Appendix S1**.

Initially, comprehensive searches were conducted in various databases in PubMed, Scopus, Web of Science, and Cochrane Library. Then, the titles and abstracts of the identified studies were screened. Additionally, a manual search was performed on the reference lists of the included studies to identify any relevant articles. The full texts of new studies were then assessed against the predetermined inclusion and exclusion criteria.

Any disagreements or discrepancies were resolved through discussions and the final judgment was by the first author. Subsequently, the included studies underwent quality assessment. Finally, the results were synthesized, and network meta-analyses were conducted to analyze and interpret the collective findings.

### 2.1 Search strategy

A literature search was performed using a concept-based approach, focusing on keywords related to “Glucagon-Like Peptide-1 Receptor,” “Dipeptidyl-Peptidase IV Inhibitors,” and “Sodium-Glucose Cotransporter 2 Inhibitors”. A comprehensive exploration was conducted electronically on four different databases, namely PubMed, Scopus, Web of Science, and Cochrane Library, encompassing the period from their commencement until the 7th of July 2023. To ensure inclusiveness, a search strategy was devised employing a blend of keywords and medical subject heading terms (MeSH). The search terms along with specific keywords showed in **Supplementary Appendix S2**.

### 2.2 Participants and inclusion criteria

Specific criteria were employed during the screening method to detect the suitability of papers for inclusion in our study following the PICOS (population, interventions, comparators, outcomes, and study designs) formatting style.

To meet the eligibility requirements, we included only real population-based cohort studies (S) relating to SGLT-2i, GLP-1 RAs, or DPP-4i utilization (I) in patients with T2DM (P) and compared them with control or other anti-diabetic agents (C) and recorded overall fractures as an outcome (O). Furthermore, English-language full-text articles were considered for inclusion. Conversely, any other article designs were omitted.

### 2.3 Outcome measures

The primary focus of our research is to examine the relationship between SGLT-2i, GLP-1 RAs, or DPP-4i utilization in patients with T2DM, and the risk of bone fracture.

### 2.4 Data extraction

The data extraction process involved using a predefined template, which included details from the trials, such as the primary investigator’s name, year of publication, sample size, duration of the trial, types of interventions employed, and control measures. Additionally, we considered the baseline characteristics of the patients, encompassing age, duration T2DM, gender, initial HbA1c levels, body mass index (BMI), and body weight. Furthermore, we examined the outcomes related to bone fractures, encompassing both overall fractures and fractures specific to the limbs and hip if present.). Two investigators extracted data independently in duplicate, and any discrepancies were resolved by discussion and consensus.

### 2.5 Assessment of risk of bias

To critically evaluate the studies involved in our research, we employed the Newcastle–Ottawa Scale (NOS) (32). It was utilized to evaluate the risk of bias in cohort studies. This tool assessed the quality of observational studies established on three essential domains: subject selection, the equivalence of individuals about demographics and critical potential confounders, and the ascertainment of the predetermined outcome. The final collective score that could be obtained by each study ranged from 0 to 9, where a score ≥7 was classified as a good-quality trial.

### 2.6 Data analysis

A network meta-analysis (NMA) had been performed using R version 4.2.2 – “Innocent and Trusting” to investigate the risk of total fractures as a primary outcome among patients who used GLP-1RAs, SGLT-2i or DPP-4i versus each other or other GLMs.

The odds ratio (OR) and 95% confidence interval (CI) had been summarized overall network and for each pairwise direct and indirect comparison. The consistency under the assumption of a full design-by-treatment interaction *random effects model* was performed to ensure the applicability of the NMA. A *node-splitting model* had been performed to evaluate the direct and indirect effect sizes inconsistency which were visualized by a forest plot. The surface under the cumulative ranking curve (SUCRA) as well as the P-scores had been calculated for each treatment in the network meta-analysis to detect their cumulative ranking probabilities in lowering the risk of total fractures.

### 2.7 Publication bias

The risk of publication bias had been checked using the comparison–adjusted funnel plot. Also, the linear regression and the rank correlation tests were used to check the funnel plot asymmetry.

## 3 Results

Initially, a comprehensive search yielded a total of 893 studies. After removing duplicate records using Endnote software (Version X8.2), the final dataset consisted of 213 studies. A title and abstract screening was then conducted on these studies to exclude reviews, animal and *in vitro* studies, and case reports. Subsequently, 115 studies were excluded, resulting in 98 papers for full-text screening and data extraction. Out of these 98 papers, 85 were excluded based on the predefined criteria established before the study commenced.

**Figure 1:**
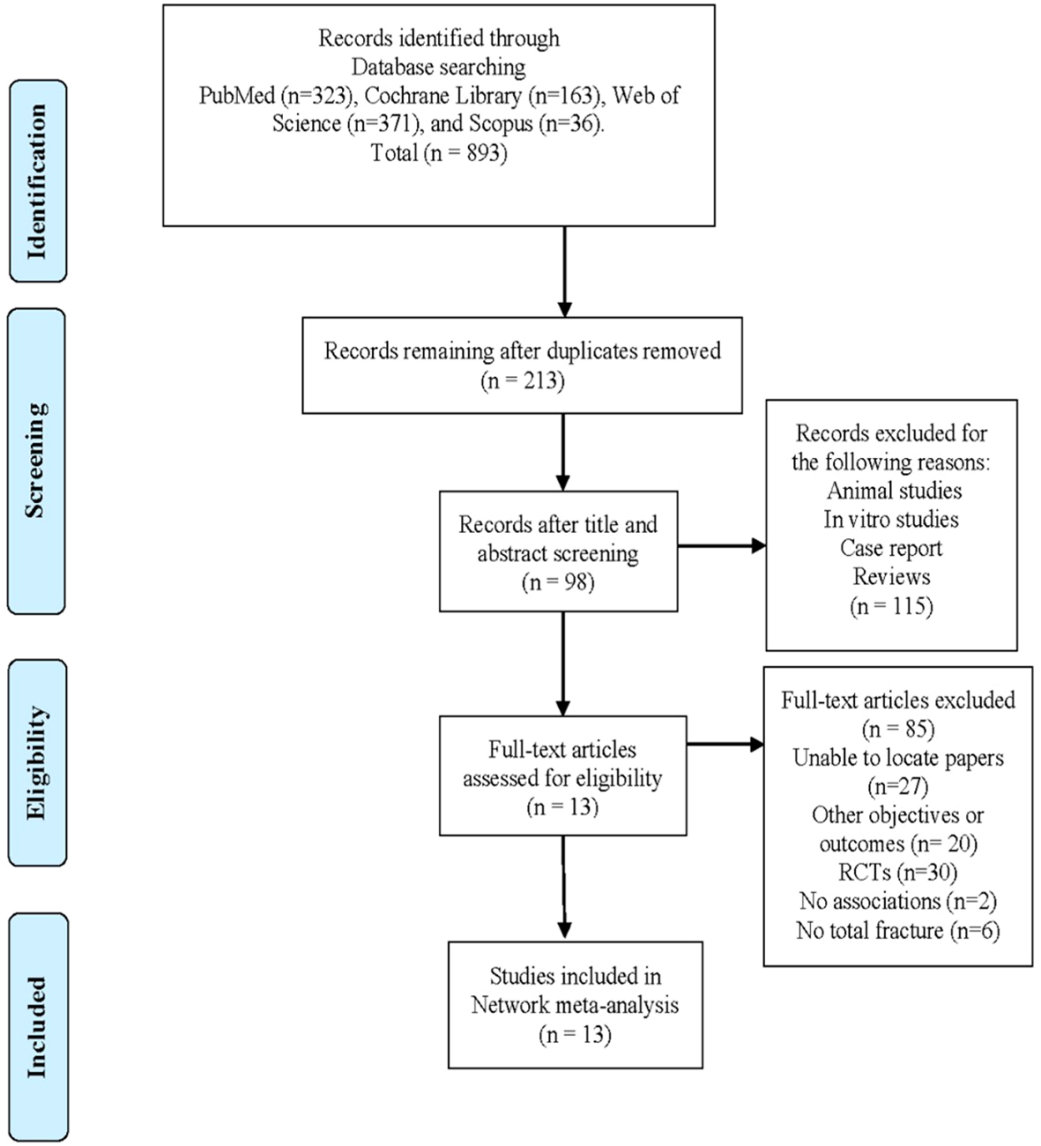
PRISMA flow diagram for the included studies.

After this rigorous screening process, the remaining 13 studies were included in the data synthesis and network meta-analysis (**Figure 1**).

### 3.1 Study characteristics

**Table 1** provides an overview of the study characteristics, including information on the participants. Overall, 13 population-based cohort studies (n = 1,064,952 patients) encountered the entitlement criteria and were involved in our NMA. Most studies were two-armed (n= 11) (12, 33–42), and two studies were three-armed (43, 44). Information concerning contributor attributes, drugs administered, and clinical investigations was gathered from files or records. The duration of follow-up varied from 9 months to 5 years. The studies included in the analysis predominantly adjusted for age and gender as the most commonly considered variables.

**Table 1:** Characteristics of the included studies.

Author name and Year	Country	Study duration	Treatment Classes	Treatment drugs	Sample size for each group	Mean age, year (SD)	Mean BMI, (kg/m2), (SD)	Obesity, n (%)	Mean HbA1c, % (SD)	Duration of T2DM (year)	Follow-up duration, Median(IQR) or Mean (SD)	Total no. of fractures n, (%) for each group
Al-Mashhadi, 2022 (a) (33)	Danmark	2012 - 2018	1.SGLT-2i group	canagliflozin, empagliflozin, or dapagliflozin	N= 13,775	60.0 (± 11.4)	NR	3,509 (25.5%)	NR	5.80 [(IQR) 2.62–9.14]	334 [139–662] Median(IQR) days	174 (1.9)
			2.GLP-1RA group	NR	N= 13,768	57.4 (± 12.1)	NR	5,373 (39.0%)	NR	5.56 [(IQR) 2.57–9.20]	497 [185–1,077] Median(IQR) days	201 (2.2)
Al-Mashhadi, 2022 (b) (34)	Danmark	2007 - 2018	GLP-1 RA group	liraglutide, semaglutide, exenatide, dulaglutide, and lixisenatide	N= 16,723	56.6 (± 12.0)	NR	6,929 (41.4%)	NR	4.84 [(IQR) 2.07–8.44]	637 [222–1,403] Median(IQR) days	647 (4.0)
			DPP-4i group	NR	N= 26,093	63.6 (± 12.4)	NR	6,058 (23.2%)	NR	4.51 [(IQR) 1.71–5.54]	519 [196–1,133] Median(IQR) days	552 (3.4)
Cowan, 2022 (35)	Canada	2015 - 2019	SGLT-2i	canagliflozin, empagliflozin, or dapagliflozin	N= 38,994	72 (5)	NR	NR	8.0 (1.5)	NR	1 year	172 (0.5)
			DPP-4i	saxagliptin, sitagliptin, or linagliptin	N= 105,700	74 (7)	NR	NR	8.1 (1.6)	NR	1 year	170 (0.4)
Driessen, 2015 (12)	United Kingdom (UK)	2007 - 2012	GLP-1 RA	liraglutide and exenatide	N = 8354	53.5 (10.5)	37.5 (7.1)	NR	8.6 (1.9)	NR	5.1 [3.6–5.2] Median(IQR) years	122 (1.5)
			Never- GLP-1 RA users	NR	N = 208,462	61.0 (15.1)	31.0 (6.5)	NR	8.0 (1.8)	NR	3.6 [1.6–5.2] Median(IQR) years	8449 (4.5)
Fralick, 2019 (36)	United States (US)	2013 -2015	SGLT-2i	Canagliflozin	N= 56 506	54.53 (9.79)	NR	NR	8.74 (1.82)	NR	264 (197) days Mean (SD)	84 (0.15)
			GLP-1 RA	Exenatide, liraglutide, albiglutide or dulaglutide	N= 56 507	54.55 (10.00)	NR	NR	8.60 (1.90)	NR	233 (188) days Mean (SD)	82 (0.14)
Han, 2021 (37)	Korea	2014 -2016	SGLT-2i	dapagliflozin, empagliflozin, or ipragliflozin	N = 15 699	71.9 (5.5)	NR	NR	NR	NR	384.7 ± 246.3 days. Mean (SD)	0.0887
			DPP-4i	sitagliptin, saxagliptin, linagliptin, vildagliptin, alogliptin, anagliptin, tenegliptin, gemigliptin, or evogliptin	N = 15 699	71.8 (5.5)	NR	NR	NR	NR	384.7 ± 246.3 days. Mean (SD)	0.0931
Lin, 2018 (38)	Taiwan	2009 - 2012	DPP-4i users	sitagliptin	N = 5,311	63 (12.4)	NR	NR	NR	8.25 (3.88) (mean, SD)	3.38 (1.19) years Mean (SD)	607 (11.4)
			Non- DPP-4i users	NR	N = 18,080	60.9 (13.8)	NR	NR	NR	1.31 (5.18) mean, SD	3.30 (1.32) years Mean (SD)	4500 (24.9)
Lui, 2023 (39)	Hong Kong	2007–2020	SGLT-2i	Canagliflozin, Dapagliflozin Empagliflozin, and Ertugliflozin	N = 14,348	60.6 (± 11.2)	27.6 (± 5.2)	NR	8.4 (± 1.6)	10.7 ± 8.5 (mean, SD)	mean 9 months	25 (0.174)
			DPP-4i	Alogliptin Linagliptin Sitagliptin Vildagliptin	N = 14,348	60.5 (± 11.7)	27.5 (± 5.5)	NR	8.4 (± 1.5)	10.7 ± 8.3 (mean, SD)	10 months (mean)	24 (0.167)
Majumdar, 2016 (40)	50 states of the US	2004 - 2009	DPP-4i Exposure	sitagliptin	N= 8894	52 (9)	NR	NR	NR	NR	2.2 years	53 (0.6)
			No DPP-4i Exposure	other antidiabetic drug	N= 63 844	52 (10)	NR	NR	NR	NR	2.2 years	688 (1.1)
Toulis, 2018 (41)	UK	2013-2016	SGLT2i group	dapagliflozin	N= 4548	59.4 (9.4)	34.7 (6.8)	NR	9.1 (3.8)	9.9 (5.8) (mean, SD)	12.9 (8.4) months Mean (SD)	58 (1.28)
			Control group	standard antidiabetic medication	N= 18 070	59.4 (9.4)	34.4 (6.6)	NR	7.5 (3.8)	9.6 (5.7)	11.9 (8.2) months Mean (SD)	231 (1.28)
Ueda, 2018 (42)	Sweden and Denmark	2013 - 2016	SGLT-2i	dapagliflozin, 61%; empagliflozin, 38%; canagliflozin, 1%	N= 17 213	61 (10)	NR	2634 (15%)	NR	NR	235 (118-426) days Median(IQR)	228 (1.32)
			GLP-1 RA		N= 17 214	61 (10)	NR	2726 (16%)	NR	NR	314 (144-591) days Median(IQR)	263 (1.53)
Zhao, 2021 (43)	Europe, North America, South America, Asia, Oceania, China	2004-2019	SGLT-2i	Canagliflozin Dapagliflozin Empagliflozin Ertugliflozin	N= 169,132 total fractures overall (not reported separately for each group).	NR	NR	NR	NR	NR	NR	317 (0.18)
			other GLMs	Metformin Glyburide Glipizide Glimepiride Exenatide Lixisenatide Liraglutide Albiglutide Dulaglutide Pioglitazone Sitagliptin Saxagliptin Alogliptin		NR	NR	NR	NR	NR	NR	2274 (1.34)
			GLMs+ SGLT-2i	Metformin + SGLT-2i, Glyburide + SGLT-2i, Glipizide + SGLT-2i, Glimepiride + SGLT-2i, Exenatide + SGLT-2i, Lixisenatide + SGLT-2i, Liraglutide + SGLT-2i, Albiglutide + SGLT-2i, Dulaglutide + SGLT-2i, Pioglitazone + SGLT-2i, Rosiglitazone + SGLT-2i, Sitagliptin + SGLT-2i, Saxagliptin + SGLT-2i, Alogliptin + SGLT-2i		NR	NR	NR	NR	NR	NR	180 (0.11)
Zhuo, 2021 (44)	US	2013 -2017	SGLT-2i	canagliflozin, dapagliflozin, or empagliflozin	N = 45 889	71.60 (4.96)	NR	NR	NR	NR	268 (262) days, Mean (median)	158 (0.34)
			DPP-4i	alogliptin, linagliptin, saxagliptin, or sitagliptin	N = 45 890	71.64 (5.13)	NR	NR	NR	NR	295 (278) days, Mean (median)	195 (0.42)
			GLP-1 RA	albiglutide, dulaglutide, exenatide, liraglutide, lixisenatide, or semaglutide)	N = 45 891	71.67 (4.97)	NR	NR	NR	NR	250 (249) days, Mean (median)	148 (0.32)

BMI, Body Mass Index; HBA1c, glycated hemoglobin test; T2DM, type-2 diabetes mellitus; DPP-4i, dipeptidyl peptidase-4 inhibitors; GLP-1 RA, glucagon-like peptide-1 receptor agonists; SGLT-2i, sodium-glucose cotransporter-2 inhibitors; GLMs, glucose-lowering medications; SD, Standard Deviation; IQR, Inter Quartile Range.

### 3.2 Study setting

The included studies were mostly conducted in wide geographical regions, spanning from 2007 to 2020. Studies were performed in the United States (US) (n=3) (36, 40, 44), Denmark (n=3) (33, 34, 42), United Kingdom (UK) (n=2) (12, 41), and one was conducted in each; Canada (35), Korea (37), Taiwan (38), Hong Kong (39), Sweden (42), Europe, North and South America, Asia, Oceania, and China (43).

### 3.3 Assessment of bias/study quality

All assessed cohort studies were of good quality (i.e., NOS scores were above 7). The Newcastle−Ottawa quality assessment criteria were available in **Supplementary Appendix S3**.

### 3.4 Network meta-analysis of DPP-4i, GLP-1 RAs, and SGLT-2i on total fracture risks

**Figure 2:**
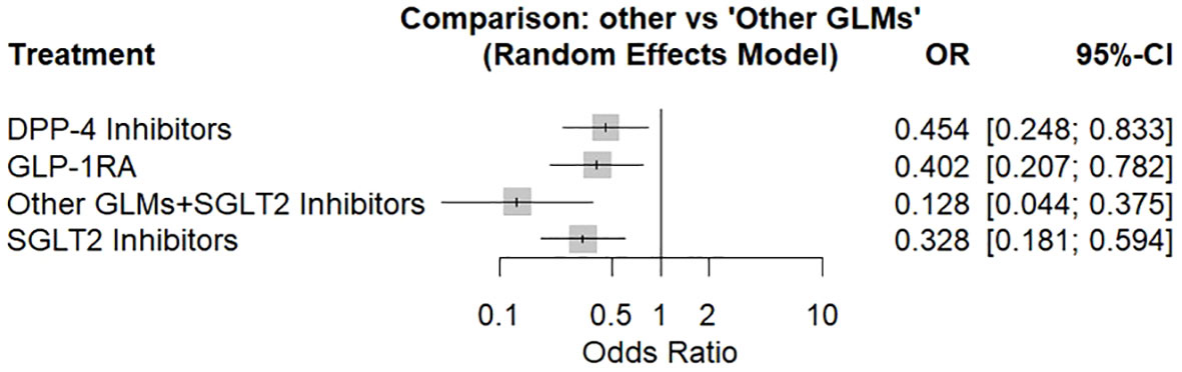
Forest plot for the risk of total fracture network meta-analysis (other GLMs as the reference). SGLT-2i, sodium-glucose cotransporter-2 inhibitors; GLMs, glucose lowering medications; GLP-1 RAs, glucagon-like peptide-1 receptor agonists; DPP-4i, dipeptidyl peptidase-4 inhibitors.

The network meta-analysis had been performed via random effects model including 13 population-based cohort studies giving the odds ratio of total bone fractures associated with each treatment in comparison to other glucose-lowering medications shown in (**Figure 2**).

There was a significant decrease in the fracture risk by about 87% associated with patients who used SGLT2 inhibitors in combination with other glucose-lowering medications, followed by SGLT2 inhibitors alone by about 67%, then GLP-1 receptor agonists by about 60%, and at last DPP-4 inhibitors by about 55%. About 98% was the total heterogeneity with 95%CI: [97.3%; 98.4%] and Q = 541.60 with a significant test of heterogeneity (p < 0.0001) and a heterogeneity variance (tau^2 = 0.363).

**Figure 3:**
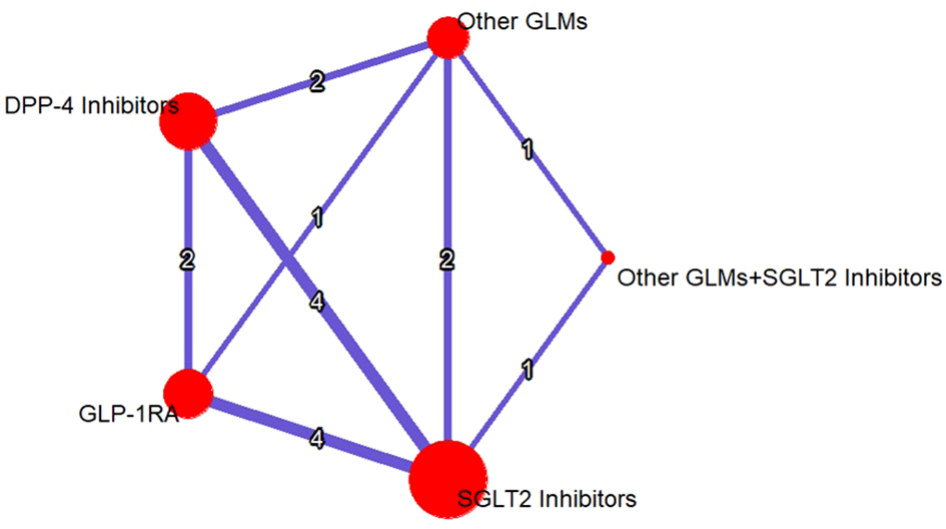
Evidence structure network meta-analysis for GLP-1RAs, SGLT-2i, DPP-4i, and other GLMs on the risk of total fractures. The number on each arm represented the number of the studies discussed in each pairwise comparison and the size of the heads were correlated to the sample size. SGLT-2i, sodium-glucose cotransporter-2 inhibitors; GLMs, glucose lowering medications; GLP-1 RAs, glucagon-like peptide-1 receptor agonists; DPP-4i, dipeptidyl peptidase-4 inhibitors.

An evidence structure for the NMA of the three classes of study treatments on the risk of total fractures was illustrated in (**Figure 3**).

**Figure 4:**
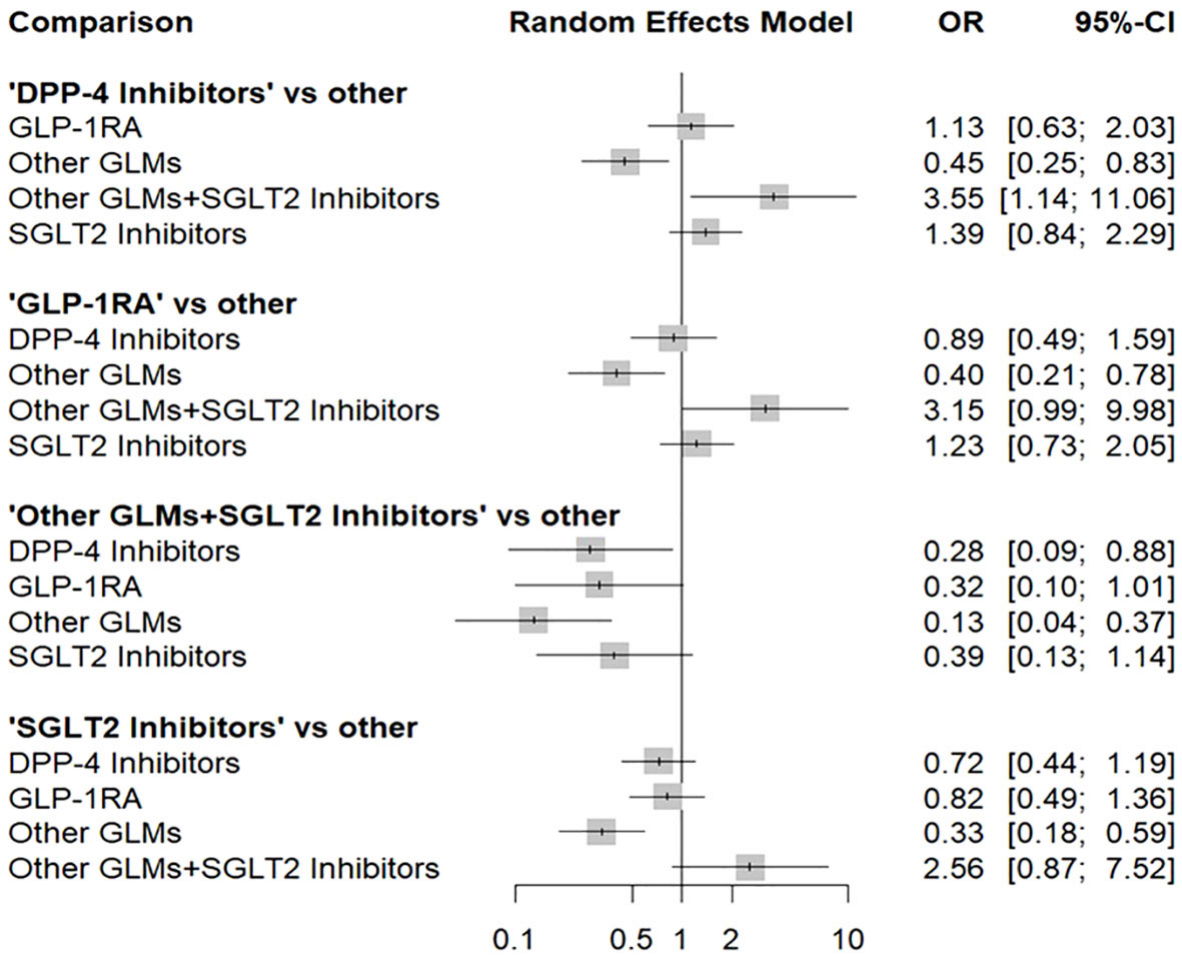
Forest plot for the risk of total fracture network meta-analysis (each treatment versus other treatments). SGLT-2i, sodium-glucose cotransporter-2 inhibitors; GLMs, glucose lowering medications; GLP-1 RAs, glucagon-like peptide-1 receptor agonists; DPP-4i, dipeptidyl peptidase-4 inhibitors.

**Figure 4** shows the total risk of fracture of each therapy compared with other therapies. DPP-4i did not increase total fracture risk compared with GLP-1 RAs (OR: 1.13, 95% CI: 0.63–2.03), other GLMs (OR: 0.45, 95% CI: 0.25–0.83), other GLMs + SGLT-2i (OR: 3.55, 95% CI: 1.14–11.06), and SGLT-2i (OR: 1.39, 95% CI: 0.84–2.29), respectively.

In addition, GLP-1 RAs did not increase fracture risk compared with DPP-4i (OR: 0.89, 95% CI: 0.49–1.59), other GLMs (OR: 0.40, 95% CI: 0.21–0.78), other GLMs + SGLT-2i (OR: 3.15, 95% CI: 0.99–9.98), and SGLT-2i (OR: 1.23, 95% CI: 0.73–2.05), respectively.

Also, other GLMs + SGLT-2i did not increase fracture risk compared with DPP-4i (OR: 0.28, 95% CI: 0.09–0.88), GLP-1 RAs (OR: 0.32, 95% CI: 0.10–1.01), other GLMs (OR: 0.13, 95% CI: 0.04–0.37), and SGLT-2i (OR: 0.39, 95% CI: 0.13–1.14), respectively.

Finally, SGLT-2i did not increase fracture risk compared with DPP-4i (OR: 0.72, 95% CI: 0.44–1.19), GLP-1 RAs (OR: 0.82, 95% CI: 0.49–1.36), other GLMs (OR: 0.33, 95% CI: 0.18–0.59), and other GLMs + SGLT-2i (OR: 2.56, 95% CI: 0.87–7.52), respectively.

### 3.5 Testing the inconsistency of the NMA

**Figure 5:**
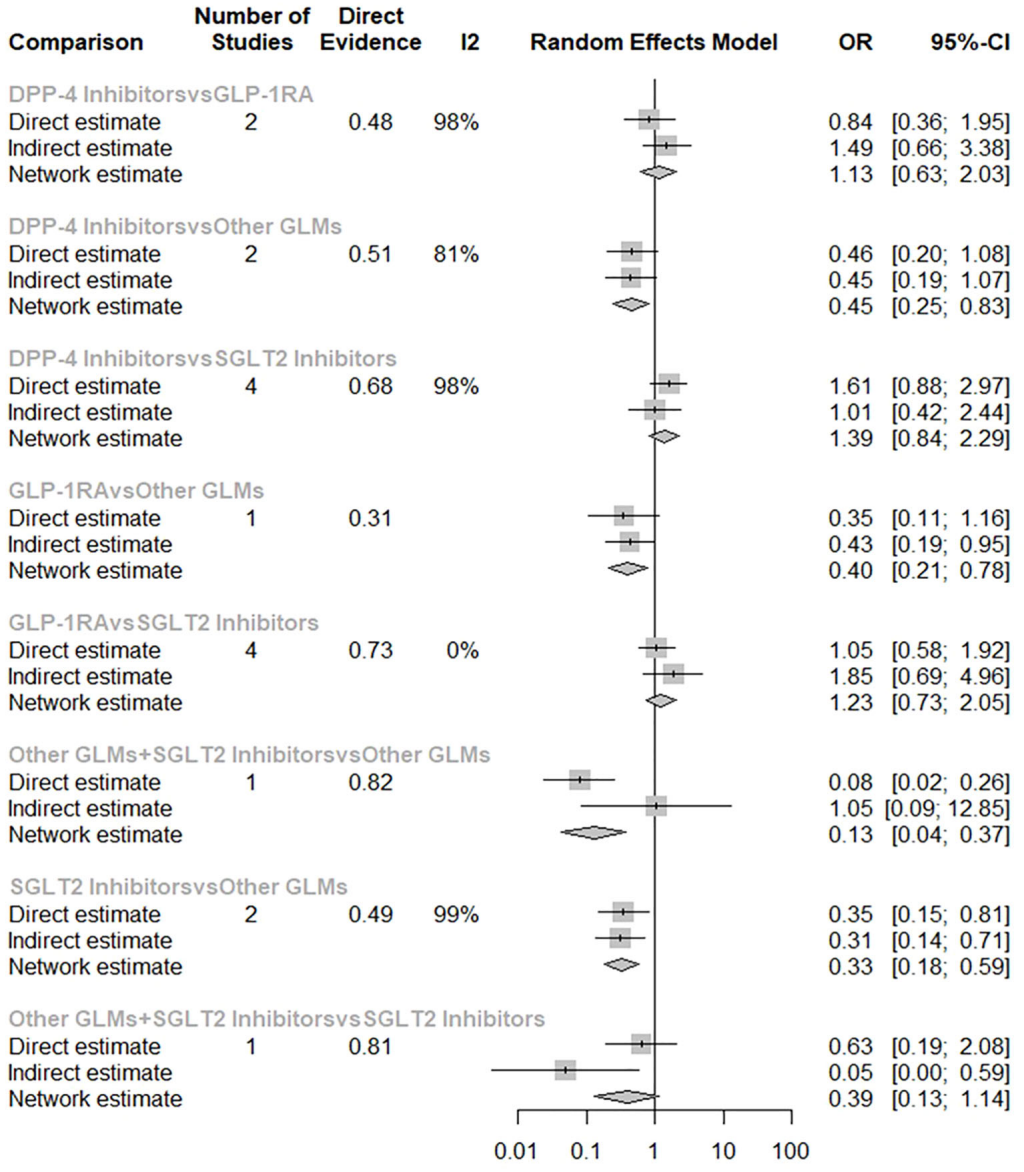
Node-splitting model separating the direct from the indirect evidence in the risk of total fractures network meta-analysis. SGLT-2i, sodium-glucose cotransporter-2 inhibitors; GLMs, glucose lowering medications; GLP-1 RAs, glucagon-like peptide-1 receptor agonists; DPP-4i, dipeptidyl peptidase-4 inhibitors.

The consistency under the assumption of a full design-by-treatment interaction random effects model between designs Q = 6.52 with non-significant p-value = 0.3678 which provided evidence for the absence of inconsistency in our NMA. In addition, the node-splitting model tests the disagreement between the direct and the indirect evidence with non-significant differences which proves the absence of any disagreement as shown in (**Figure 5**).

The league table for the random effects estimates resulting from the network meta-analysis for the risk of total fractures using pairwise comparisons can be found in **Table 2**.

**Table 2:** A league table for the random effects estimates (Odds ratios (OR) and 95%CI) resulted from the network meta-analysis for the risk of total fractures from pairwise comparisons.

V1	V2	V3	V4	V5
Other GLMs+SGLT2 Inhibitors	0.63 (0.53; 0.75)	.	.	0.08 (0.07; 0.09)
0.35 (0.30; 0.41)	SGLT2 Inhibitors	0.80 (0.74; 0.85)	0.93 (0.83; 1.03)	0.17 (0.15; 0.19)
0.27 (0.23; 0.32)	0.78 (0.73; 0.82)	DPP-4 Inhibitors	0.66 (0.59; 0.73)	0.40 (0.37; 0.44)
0.23 (0.20; 0.27)	0.66 (0.61; 0.71)	0.85 (0.78; 0.91)	GLP-1RA	0.35 (0.29; 0.42)
0.09 (0.07; 0.10)	0.24 (0.23; 0.26)	0.31 (0.29; 0.34)	0.37 (0.34; 0.41)	Other GLMs

The upper triangle of the outcome presentation included the findings of direct comparisons, where the estimation was determined by comparing the treatment indicated in the row with the treatment indicated in the column. Conversely, the lower triangle of the outcome presentation included the network meta-analysis results, where the estimation was calculated by comparing the treatment indicated in the column with the treatment indicated in the row. SGLT-2i, sodium-glucose cotransporter-2 inhibitors; GLMs, glucose lowering medications; GLP-1 RAs ,glucagon-like peptide-1 receptor agonists; DPP-4i, dipeptidyl peptidase-4 inhibitors.

The SUCRA values showed a probability of more than 97% for the combination of SGLT2 Inhibitors with other GLMs to be the best treatment decreasing the risk of total fractures followed by 68% for SGLT2 Inhibitors alone and about 48% for GLP-1RA and about 36% for DPP-4 Inhibitors compared to other glucose-lowering medications which showed the lowest ranking. Also, the P-scores showed very similar values for the SUCRA ones (**Table 3**).

**Table 3:** Ranking of treatments effects on the risk of total fractures.

Study Treatments	SUCRA	P-scores
Other GLMs+SGLT2 Inhibitors	0.9728	0.9791
SGLT2 Inhibitors	0.6825	0.6810
GLP-1RA	0.4790	0.4744
DPP-4 Inhibitors	0.3638	0.3633
Other GLMs	0.0020	0.0023

SGLT-2i, sodium-glucose cotransporter-2 inhibitors; GLMs, glucose lowering medications; GLP-1 RAs, glucagon-like peptide-1 receptor agonists; DPP-4i, dipeptidyl peptidase-4 inhibitors.

**Figure 6:**
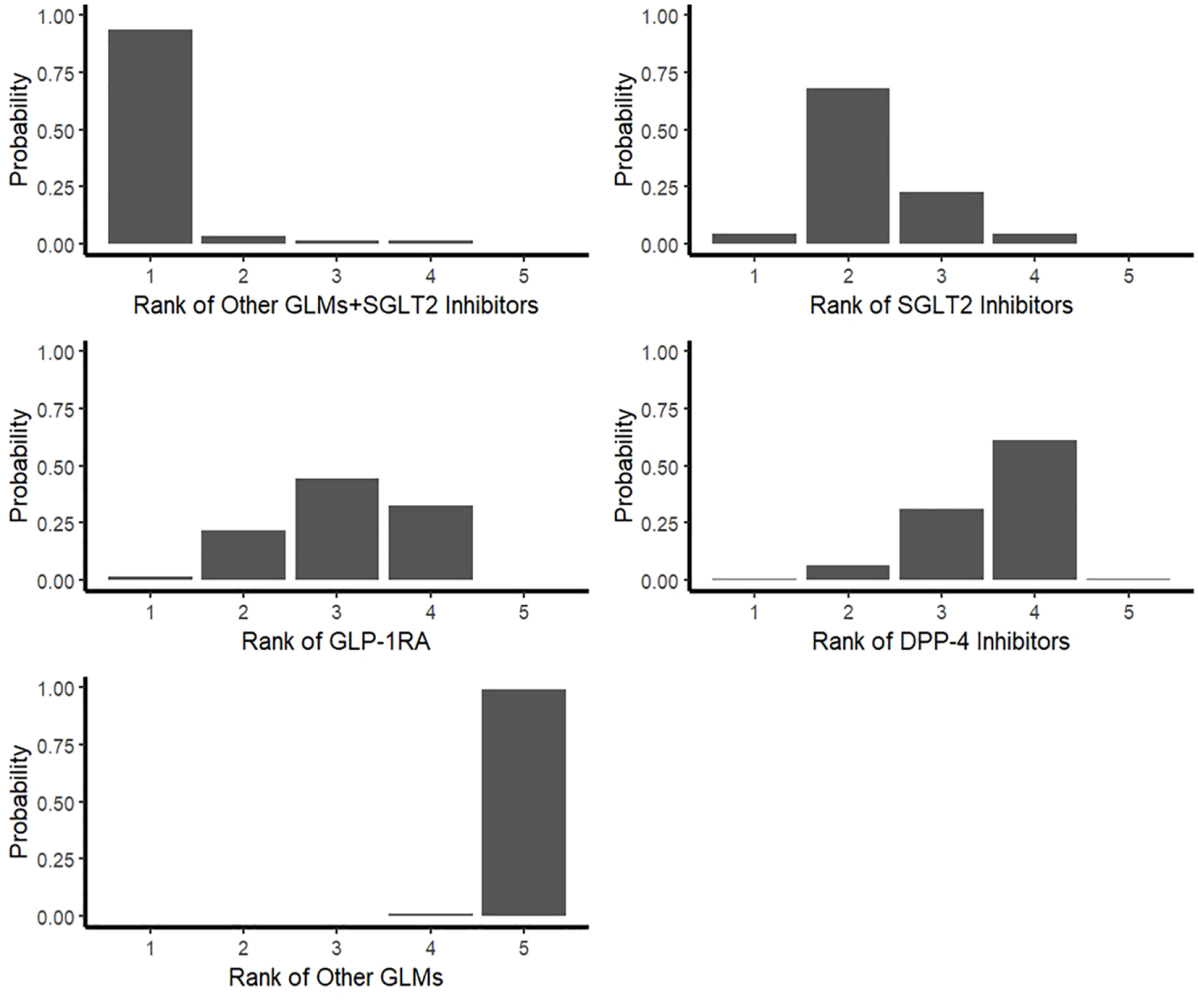
Rankogram for the risk of total fractures network meta-analysis. SGLT-2i, sodium-glucose cotransporter-2 inhibitors; GLMs, glucose lowering medications; GLP-1 RAs, glucagon-like peptide-1 receptor agonists; DPP-4i, dipeptidyl peptidase-4 inhibitors.

Furthermore, the rankogram random effects model based on 1000 simulations showed that the combination of SGLT2 Inhibitors with other GLMs had a probability of 93% to be the most therapy associated with decreasing the risk of total fractures and SGLT2 Inhibitors alone also had low fracture risk by about 73% probability. However, GLP-1RA had a low fracture risk by about 67% probability (**Table 4**; **Figure 6**).

**Table 4:** Rankogram random effects model.

Study Treatments	1	2	3	4	5
DPP-4 Inhibitors	0.005	0.067	0.311	0.612	0.005
GLP-1RA	0.015	0.215	0.444	0.323	0.003
Other GLMs	0	0	0	0.008	0.992
Other GLMs+SGLT2 Inhibitors	0.934	0.036	0.017	0.013	0
SGLT2 Inhibitors	0.046	0.682	0.228	0.044	0

SGLT-2i, sodium-glucose cotransporter-2 inhibitors; GLMs, glucose lowering medications; GLP-1 RAs, glucagon-like peptide-1 receptor agonists; DPP-4i, dipeptidyl peptidase-4 inhibitors.

### 3.6 Risk of publication bias

**Figure 7:**
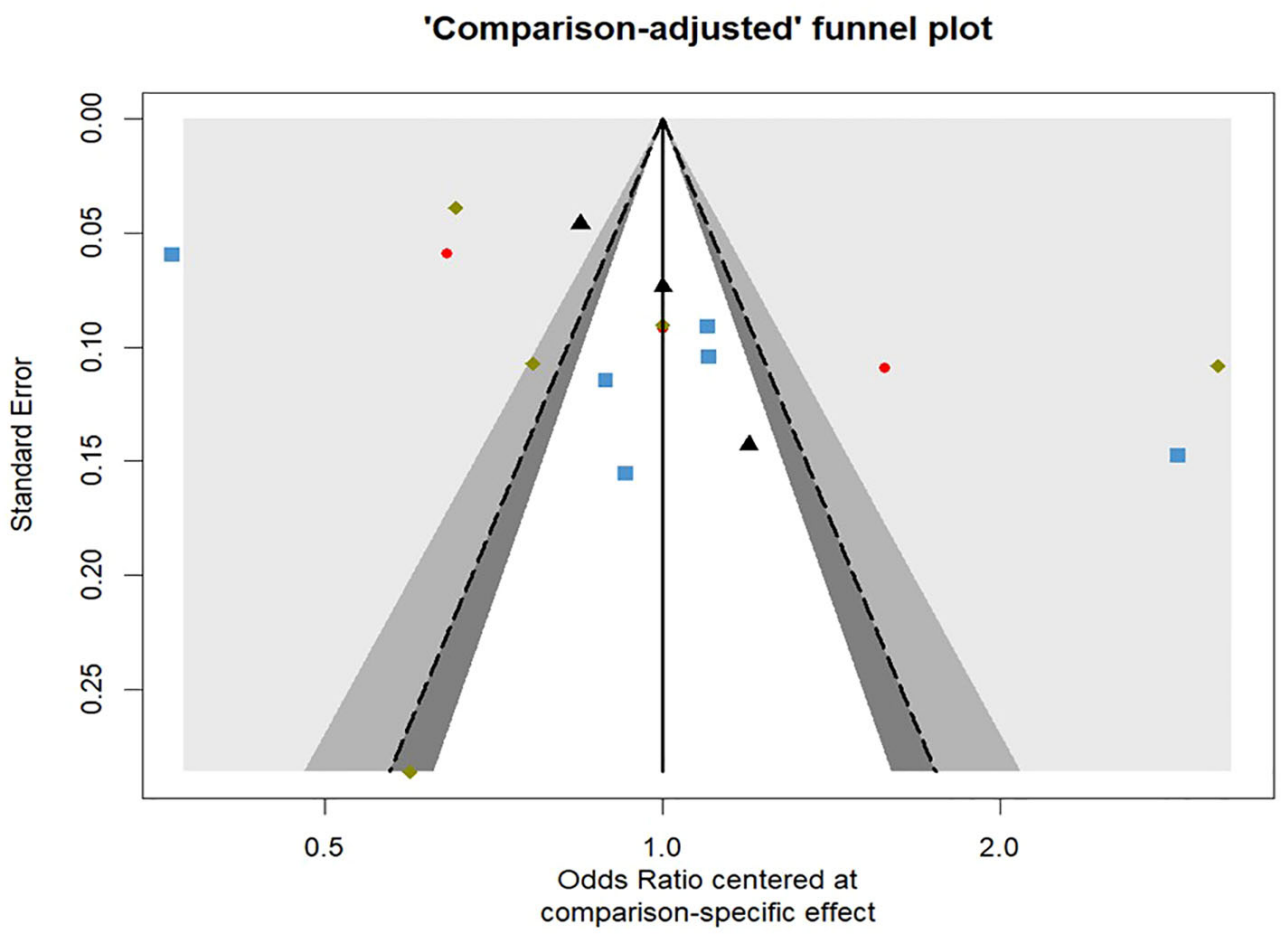
Comparison-adjusted contour enhanced funnel plot for the risk of total fractures network meta-analysis.

Funnel plots were shown in **Figure 7**. For total fracture, the linear regression test as well as the rank correlation test of funnel plot asymmetry showed non-significant results (p = 0.4804 & p = 0.2165, respectively) proving that there is no evidence for the presence of any publication bias.

## 4 Discussion

In the present population-based network meta-analysis, we analyzed 13 cohort studies, including 1,064,952 patients. The analysis is designed to explore and compare the effects of relatively recent classes of anti-diabetic drugs (SGLT-2i, GLP-1RA, or DPP-4i) on the risk of bone fracture in patients with type 2 diabetes in the real world.

There was a significant decrease in the fracture risk by about 87% associated with patients who used SGLT2 inhibitors in combination with other glucose-lowering medications, followed by SGLT2 inhibitors alone by about 67%, then GLP-1 receptor agonists by about 60%, and at last DPP-4 inhibitors by about 55%.

Through a comprehensive evaluation involving direct comparisons, indirect comparisons, and assessment of inconsistency, we obtained significant findings regarding the correlation between the utilization of anti-diabetic medications and a reduced risk of fractures.

The potential mechanisms contributing to the effect of these drugs were studied in multiple research articles. To begin with, SGLT-2 inhibitors could have a temporary impact on calcium and phosphorus homeostasis where SGLT-2 inhibition promotes phosphate reabsorption in the proximal tubule through the sodium–phosphate cotransport to compensate for renal loss of sodium along with glucose (45). Moreover, theoretically, SGLT-2 inhibitors may prompt dehydration, because they raise the overall risk of fractures by causing osmotic diuresis, intravascular volume contraction, orthostatic hypotension, and an increased risk of falls (46).

GLP-1 RA on the other hand have different proposed mechanisms. These include reducing the accumulation of advanced glycation end products (AGE), stimulating GLP-1 receptors of osteoblasts, regulating β-catenin signal transduction, and increasing the expression of osteoprotegerin genes which after certain pathways activation induces the activation, proliferation, and differentiation of osteoblasts, while causing the inhibition of osteoclasts, and bone mass formation (45).

The impact of DPP-4i on bone health and metabolism is composite and multifaceted. First of all, they affect bone metabolism through their substrates and through altering a vitamin D-linked pathway (which induces bone growth and bone remodeling) (47, 48). The quantity and functionality of osteoblasts are directly reduced by AGE buildup or AGE/RAGE (advanced glycation end products/advanced glycation end product receptors) imbalance (7). Additionally, the effect is mediated by DPP-4-related energy metabolism through the reduction of ghrelin and p38 mitogen-activated protein kinase and the increase in insulin, adiponectin, amylin, and preptin, which lowers the development of osteoclasts (47, 48).

Based on the collective evidence derived from descriptive studies and RCTs, it is established that the administration of SGLT2i, GLP-1ra, and DPP-4i is generally associated with neutral effects on fracture risk. These findings hold for homogeneous patient groups in the tightly regulated conditions of RCTs, as well as for diverse patient populations in real-world observational settings (28, 49).

The absence of this association in both descriptive studies and RCTs is reinforced by compelling proof indicating that these anti-diabetic drugs exert a slight influence on bone metabolism or bone mineral density (BMD) markers in human subjects (50–52).

Observational studies conducted in real-world settings revealed a notable trend towards a reduced fracture risk associated with GLP-1ra, or DPP-4i utilization, although this relation didn’t statistically significant (28). These results hold considerable clinical significance as other generally administrated second and third-line anti-diabetic drugs (such as insulin, thiazolidinediones, and sulfonylureas) are linked to a rise in fracture risk directly or indirectly (53–55). Consequently, considering SGLT2i, or DPP-4i as potential alternatives to those drugs can have significant implications in clinical practice.

The impact of GLP-1 receptor agonists seemed to be influenced by the particular subtype of medication employed. An investigation incorporating multiple RCTs demonstrated the link of the utilization of GLP-1ra to fracture risk, which varied depending on the specific GLP-1ra type utilized. Specifically, exenatide was linked to an increased risk of fractures, while liraglutide was associated with a decreased risk of fractures (15).

In contrast, a different meta-analysis exploring the same subject matter discovered that exenatide was linked to a decreased risk of fractures. However, this relation didn’t present with semaglutide, liraglutide, lixisenatide, albiglutide, and dulaglutide (7). The varying conclusions observed across these research analyses could be attributed to factors such as the few fracture cases examined in our investigation and the duration of the studies conducted.

However, when examining population-based cohort studies, it was found that exenatide and liraglutide did not significantly impact fracture risk (28). Similarly, RCTs imitative evidence specified that neither exenatide nor liraglutide affected BMD significantly (50, 56, 57).

A comprehensive 51 RCTs meta-analysis revealed the absence of significant link of DPP-4 inhibitors utilization to the fracture rate when compared to either a placebo or an active comparator (58). In line with these findings, Fu et al. (2016) (59) determined that the utilization of DPP-4 inhibitors had no significant impact on the risk of bone fractures among individuals with T2DM when compared to placebo or alternative anti-diabetic medications (RR = 0.95; 95% CI: 0.83–1.10).

In the comprehensive evaluation known as the Canagliflozin Cardiovascular Assessment Study (CANVAS) Program, a notable rise in fracture occurrences was observed among individuals treated with canagliflozin (4.0%) compared to those who received a placebo (2.6%). Moreover, the fracture rate was increased in the canagliflozin group (2.7%) than the non-canagliflozin one (1.9%) within the entire study population (27). Numerous RCTs meta-analyses indicated that the use of canagliflozin didn’t display a significant association with fracture risk (60–62). The inconsistency in the findings could potentially be attributed to various factors, including the duration of SGLT-2 inhibitor use with or without thiazolidinediones (TZDs) utilization, a recognized factor associated with raised fracture risk (63, 64).

Nevertheless, multiple meta-analyses and the aggregated findings from the most recent real-world meta-analysis have consistently demonstrated that SGLT-2i utilization doesn’t heighten the bone fracture risk in patients with T2DM compared to control (16, 60, 61). Hence, SGLT-2i can be regarded as a viable option in the management of diabetes for patients who are prone to fractures.

Various investigations exploring the influence of SGLT-2i, GLP-1RA, or DPP-4i on fracture risk in individuals with T2DM have suggested that the variation in gender may not relate to the elevated risk of fractures (13, 26, 65, 66). Consequently, this particular study did not specifically analyze the influence of gender factors.

It is noteworthy to discuss the frailty syndrome as it has been proven to have a crucial significance in personalized medicine in older patients (67). The assessment and incorporation of frailty status can facilitate a more personalized, patient-centered approach to care. The identification of frail elderly patients with T2DM may necessitate more stringent monitoring, vigilant follow-up, and the prioritization of interventions that correspond to their unique requirements, including but not limited to nutritional support, and tailored medication (46). However, a wider range of treatment choices may be taken into consideration for elderly patients who are considered robust or pre-frail, balancing glycemic control with the preservation of physical function and quality of life (68). In a study by Paterni et al. (67), the combination of diabetes and frailty led to a worsening of the 1-year prognosis after orthopedic surgery, specifically in frail patients. Furthermore, frailty appeared as an independent mortality predictor for individuals with HbA1c levels above 48 mmol/mol. However, in patients with strict glycemic control, there was no difference in mortality between robust and frail individuals, indicating that hypoglycemia in older patients has a negative impact regardless of their clinical status (67). Healthcare professionals can customize their management strategies to the unique needs of older adults with T2DM by incorporating the evaluation of frailty into the clinical decision-making process. This will ultimately improve patient outcomes and encourage a more comprehensive, person-centered approach to care.

This NMA consumes several strengths. The present network meta-analysis provides appreciated findings of the link between the use of relatively recent anti-diabetic drugs (SGLT-2i, GLP-1RA, or DPP-4i) and the lower risk of bone fracture by integrating data from 1,064,952 patients in the mostly wide geographical distributed population. So, this conclusion goes better than other meta-analyses of RCTs and observational studies findings, which allows the generalization of the findings to the population.

In addition, our analysis is a network meta-analysis that provides comparative assessments of various interventions within the network, delivering more precise estimations compared to single direct or indirect estimates. Additionally, it enables the determination of the ranking and hierarchy of interventions. Moreover, we calculated SUCRA as well as the P-scores for each treatment in the network meta-analysis to detect their cumulative ranking probabilities in lowering the risk of total fractures.

Finally, the majority of the studies we incorporated contained data about several potentially significant covariates, including the duration of diabetes mellitus (DM), the specific type of SGLT-2i, GLP-1RA, and DPP-4i utilized, past incidents of falls, frailty, osteoporosis, and fractures, as well as modifiable lifestyle factors such as smoking, alcohol consumption, and BMI.

However, our analysis challenged several limitations. First, given the inherent characteristics of descriptive studies, the results obtained from the current meta-analysis may have been influenced by factors such as lag or immortal time bias, as well as outstanding and unmeasured confounding variables. Additionally, the studies included in the analysis did not provide data on crucial covariates, including the severity of DM, the initiation date of medication use, prescribed dosage regimens, and biochemical parameters or markers (such as bone mineral density, turnover, formation, and resorption markers).

Second, we can’t identify possible effect convertors and heterogeneity sources due to the limited number of studies included and the lack of comprehensive subgroup analyses.

Third, the current meta-analysis relied solely on comparing individuals who were exposed to medications with those who were not, rather than utilizing more detailed and informative measures such as medication adherence, cumulative dose exposure, average daily dose, and continuous duration of use. Since most of the studies employed prescription data as a proxy for medication exposure, the results may have been influenced by a null association due to primary or secondary non-adherence. Finally, it is worth noting that fracture events were rarely confirmed through radiographic imaging.

## 5 Conclusion and future directions

By synthesizing the findings from these diverse studies, our analysis provides valuable insights into the link between the use of new anti-diabetic drugs and the lower risk of bone fracture in type 2 diabetic patients. We observe that SGLT2 inhibitors with other glucose-lowering medications combination, SGLT2 inhibitors alone, GLP-1 receptor agonists, and DPP-4 inhibitors significantly decreased the total fracture risk respectively. This population-based analysis offers the best available evidence and might be helpful for clinicians in the decision of the most suitable T2DM treatment strategies, especially for elderly patients.

There is a crucial need for additional clinical trials to explore the relationship between the utilization of anti-diabetic medications and the occurrence of bone fractures, as these fragility fractures can have a significant impact on individuals with diabetes. Furthermore, it is imperative to conduct further prospective research and randomized clinical trials to investigate this association specifically among high-risk patients with T2DM who are at a heightened risk of fractures, such as those with advanced age, low BMD, a history of fractures or osteoporosis, patients with diabetic complications and frail individuals.

As for patient management-related implications, the observed differences in fracture risk associated with various anti-diabetic medications highlight the need for closer monitoring and individualized therapy selection in this patient population. When selecting anti-diabetic medications for their patients, clinicians should take into account the patient’s fracture risk profile, which can be impacted by age, duration of diabetes, patient’s history of fractures, and other concurrent drugs. Patients diagnosed as having a higher risk of fractures may benefit from more frequent bone health check-ups, calcium and vitamin D supplements, and the use of drugs with a favorable skeletal safety profile. Additionally, as noted by our results, SGLT2 inhibitors with other glucose-lowering medications combination would seem the most appropriate choice followed by SGLT2 inhibitors alone. In contrast, a greater variety of anti-diabetic drugs may be suitable for patients with a lower risk of fracture. This personalized approach to monitoring and individualized therapy selection based on fracture risk profiles can help reduce the risk of treatment-associated fractures and improve the overall care and quality of life for patients with T2DM.

## Data Availability

The original contributions presented in the study are included in the article/**Supplementary Material**. Further inquiries can be directed to the corresponding author.
