# Supplementary material for: Risk of bone fracture by using dipeptidyl peptidase-4 inhibitors, glucagon-like peptide-1 receptor agonists, or sodium-glucose cotransporter-2 inhibitors in patients with type 2 diabetes mellitus: a network meta-analysis of population-based cohort studies
Source: Front Endocrinol (Lausanne). 2024 Oct 11;15:1410883. doi: 10.3389/fendo.2024.1410883 (PMC11502341; doi:10.3389/fendo.2024.1410883)
Supplement: Supplementary file 1 [file DataSheet1.docx]

**PRISMA NMA Checklist of Items to Include When Reporting A Systematic Review Involving a Network Meta-analysis**

| **Section/Topic** | **Item #** | **Checklist Item** | **Reported on Section** |
| --- | --- | --- | --- |
| **TITLE** | | |  |
| Title | 1 | Identify the report as a systematic review *incorporating a*  *network meta-analysis (or related form of meta-analysis).* | Title page |
| **ABSTRACT** |  |  | Title page |
| Structured summary | 2 | Provide a structured summary including, as applicable:  **Background:** main objectives  **Methods:** data sources; study eligibility criteria, participants, and interventions; study appraisal; and *synthesis methods, such as network meta-analysis.*  **Results:** number of studies and participants identified; summary estimates with corresponding confidence/credible intervals; *treatment rankings may also be discussed. Authors may choose to summarize pairwise comparisons against a chosen treatment included in their analyses for brevity.*  **Discussion/Conclusions:** limitations; conclusions and implications of findings.  **Other:** primary source of funding; systematic review registration number with registry name. |  |
| **INTRODUCTION** |  |  |  |
| Rationale | 3 | Describe the rationale for the review in the context of what is  already known*, including mention of why a network meta- analysis has been conducted.* | Introduction |
| Objectives | 4 | Provide an explicit statement of questions being addressed, with reference to participants, interventions, comparisons, outcomes, and study design (PICOS). | Introduction |
| **METHODS** |  |  |  |
| Protocol and registration | 5 | Indicate whether a review protocol exists and if and where it can be accessed (e.g., Web address); and, if available, provide  registration information, including registration number. | Methods |
| Eligibility criteria | 6 | Specify study characteristics (e.g., PICOS, length of follow-up) and report characteristics (e.g., years considered, language, publication status) used as criteria for eligibility, giving rationale. *Clearly describe eligible treatments included in the treatment network, and note whether any have been clustered*  *or merged into the same node (with justification).* | under subheading 2.2.Participants and inclusion criteria |
| Information sources | 7 | Describe all information sources (e.g., databases with dates of  coverage, contact with study authors to identify additional studies) in the search and date last searched. | under subheading 2.1.Search Strategy |
| Search | 8 | Present full electronic search strategy for at least one database,  including any limits used, such that it could be repeated. | Supplementary appendix S2 |
| Study selection | 9 | State the process for selecting studies (i.e., screening, eligibility, included in systematic review, and, if applicable, | under subheading 2.2.Participants and inclusion criteria |

|  |  | included in the meta-analysis). | |  |
| --- | --- | --- | --- | --- |
| Data collection | 10 | Describe method of data extraction from reports (e.g., piloted | |  |
| process |  | forms, independently, in duplicate) and any processes for | | under subheading 2.4. Data Extraction |
|  |  | obtaining and confirming data from investigators. | |  |
| Data items | 11 | List and define all variables for which data were sought (e.g., | |  |
|  |  | PICOS, funding sources) and any assumptions and | | under subheading 2.3. Outcome measures |
|  |  | simplifications made. | |  |
| **Geometry of the network** | **S1** | Describe methods used to explore the geometry of the  treatment network under study and potential biases related to it. | | under subheading 2.6. Data analysis |
|  |  | This should include how the evidence base has been | |  |
|  |  | graphically summarized for presentation, and what | |  |
|  |  | characteristics were compiled and used to describe the evidence | |  |
|  |  | base to readers. | |  |
| Risk of bias within | 12 | Describe methods used for assessing risk of bias of individual | |  |
| individual studies |  | studies (including specification of whether this was done at the | | under subheading 2.7. Publication bias |
|  |  | study or outcome level), and how this information is to be used | |  |
|  |  | in any data synthesis. | |  |
| Summary measures | 13 | State the principal summary measures (e.g., risk ratio, | |  |
|  |  | difference in means). *Also describe the use of additional* | | under subheading 2.6. Data analysis |
|  |  | *summary measures assessed, such as treatment rankings and* | |  |
|  |  | *surface under the cumulative ranking curve (SUCRA) values,* | |  |
|  |  | *as well as modified approaches used to present summary* | |  |
|  |  | *findings from meta-analyses.* | |  |
| Planned methods of | 14 | Describe the methods of handling data and combining results of | |  |
| analysis |  | studies for each network meta-analysis. This should include, | | under subheading 2.6. Data analysis |
|  |  | but not be limited to:   - *Handling of multi-arm trials;* | |  |
|  |  | - *Selection of variance structure;* - *Selection of prior distributions in Bayesian analyses;* | | |
|  |  | *and*   - *Assessment of model fit.* | | |
| **Assessment of** | **S2** | Describe the statistical methods used to evaluate the agreement | | |
| **Inconsistency** |  | of direct and indirect evidence in the treatment network(s) | studied. Describe efforts taken to address its presence when | |
|  |  | found. **under subheading 2.6. Data analysis** | | |
| Risk of bias across | 15 | Specify any assessment of risk of bias that may affect the | | |
| studies |  | cumulative evidence (e.g., publication bias, selective reporting | | |
|  |  | within studies).  **under subheading 2.7. Publication bias** | | |
| Additional analyses | 16 | Describe methods of additional analyses if done, indicating | | |
|  |  | which were pre-specified. This may include, but not be limited | | |
|  |  | to, the following: | | |
| - Sensitivity or subgroup analyses; - Meta-regression analyses; - *Alternative formulations of the treatment network; and* - *Use of alternative prior distributions for Bayesian analyses (if applicable).*   **under subheading 2.6. Data analysis** | | | | |

| **RESULTS†** |  |  | | |  |
| --- | --- | --- | --- | --- | --- |
| Study selection | 17 | Give numbers of studies screened, assessed for eligibility, and **Heading 3. Results**  included in the review, with reasons for exclusions at each stage,  ideally with a flow diagram. | | |  |
| **Presentation of network structure** | **S3** | Provide a network graph of the included studies to enable  visualization of the geometry of the treatment network. **Figures 2-4** | | |  |
| **Summary of network geometry** | **S4** | Provide a brief overview of characteristics of the treatment  **NA**  network.  This may include commentary on the abundance of trials and  randomized patients for the different interventions and  pairwise comparisons in the network, gaps of evidence in  the treatment network, and potential biases reflected  by the network structure. | | |  |
| Study characteristics | 18 | For each study, present characteristics for which data were  **Subheading**  **3.1.Study characteristics**  extracted (e.g., study size, PICOS, follow-up period) and  provide the citations. | | |  |
| Risk of bias within studies | 19 | Present data on risk of bias of each study and, if available,  any outcome level assessment.  **Subheading 3.3. Assessment of bias/study quality** | | |  |
| Results of individual studies | 20 | For all outcomes considered (benefits or harms), present, for each study: **Table 1**   1. simple summary data for each intervention group, 2. and effect estimates and confidence intervals.   *Modified approaches may be needed to deal with information*  *from larger networks.* | | |  |
| Synthesis of results | 21 | **Subheading 3.4.Network Meta-Analysis**  **of DPP-4i, GLP-1 RAs,**  **and SGLT-2i on**  **Total Fracture Risks**  Present results of each meta-analysis done, including confidence/  credible intervals.  *In larger networks, authors may focus on comparisons versus*  *a particular comparator (e.g. placebo or standard care),*  *with full findings presented in an appendix.*  *League tables and forest plots may be considered to*  *summarize pairwise comparisons.*  If additional summary measures were explored  (such as treatment rankings), these  should also be presented. | | |  |
| **Exploration for inconsistency** | **S5** | Describe results from investigations of inconsistency. This may include such information as measures of model fit to compare consistency and inconsistency models, *P* values from statistical tests, or summary of inconsistency estimates from different  parts of the treatment network.  **Subheading 3.5.Testing the**  **inconsistency of the NMA** | | |  |
| Risk of bias across studies | 22 | Present results of any assessment of risk of bias across studies for the evidence base being studied.  **Subheading 3.3.Assessment of bias/study quality** | | |  |
| Results of additional analyses | 23 | Give results of additional analyses, if done (e.g., sensitivity or subgroup analyses, meta-regression analyses*, alternative network geometries studied, alternative choice of prior distributions for Bayesian analyses,* and so forth).  **Subheading 3.6.Publication bias** | | |  |
| **DISCUSSION** |  |  | | |  |
| Summary of evidence | 24 | Summarize the main findings, including the strength of evidence for each main outcome; consider their relevance to key groups (e.g., healthcare providers, users, and policy makers. | | **Discussion** |  |
| Limitations | 25 | Discuss limitations at study and outcome level (e.g., risk of bias), and at review level (e.g., incomplete retrieval of identified research, reporting bias). *Comment on the validity of* | *the assumptions, such as transitivity and consistency. Comment*    **Last section of**  **discussion** | |  |

| *on any concerns regarding network geometry (e.g., avoidance of certain comparisons).* | | | |
| --- | --- | --- | --- |
| Conclusions | 26 | Provide a general interpretation of the results in the context of other evidence, and implications for future research. | **Conclusion**  **And**  **Future**  **Directions** |
| **FUNDING** |  |  | **Funding** |
| Funding | 27 | Describe sources of funding for the systematic review and other support (e.g., supply of data); role of funders for the systematic review. This should also include information regarding whether funding has been received from manufacturers of treatments in the network and/or whether some of the authors are content experts with professional conflicts of interest that could affect  use of treatments in the network. |  |

PICOS = population, intervention, comparators, outcomes, study design.

* Text in italics indicateS wording specific to reporting of network meta-analyses that has been added to guidance from the PRISMA statement.

† Authors may wish to plan for use of appendices to present all relevant information in full detail for items in this section.
