# Supplementary material for: Risk of bone fracture by using dipeptidyl peptidase-4 inhibitors, glucagon-like peptide-1 receptor agonists, or sodium-glucose cotransporter-2 inhibitors in patients with type 2 diabetes mellitus: a network meta-analysis of population-based cohort studies
Source: Front Endocrinol (Lausanne). 2024 Oct 11;15:1410883. doi: 10.3389/fendo.2024.1410883 (PMC11502341; doi:10.3389/fendo.2024.1410883)

**Search strategy**

**Of**

**Risk of Bone Fracture by Using Dipeptidyl Peptidase-4 Inhibitors, Glucagon-like Peptide-1 Receptor Agonists, or Sodium-Glucose Cotransporter-2 Inhibitors in Patients with Type 2 Diabetes Mellitus: A Network Meta-analysis of population-based cohort studies**

1. Breaking into keywords (PICO):

| **Population** | **Intervention** | **Control** | **Outcome** |
| --- | --- | --- | --- |
| Patients with Type 2 Diabetes Mellitus | Dipeptidyl Peptidase-4 Inhibitors  And  Glucagon-like Peptide-1 Receptor Agonists  And  Sodium-Glucose Cotransporter-2 Inhibitors | Other glucose-lowering drugs | Bone Fracture |

1. Finding relevant keywords with linking words:

| **Keywords** | **Relevant** |
| --- | --- |
| Dipeptidyl Peptidase-4 Inhibitors (DPP-4i) | Dipeptidyl Peptidase-4 Inhibitors OR DPP-4i OR sitagliptin OR vildagliptin OR saxagliptin OR linagliptin OR anagliptin OR teneligliptin OR alogliptin OR trelagliptin OR gemigliptin OR dutogliptin OR omarigliptin |
| Glucagon-like Peptide-1 Receptor Agonists (GLP-1ra) | Glucagon-like Peptide-1 Receptor Agonists OR GLP-1ra OR exenatide OR liraglutide OR lixisenatide OR albiglutide OR dulaglutide |
| Sodium-Glucose Cotransporter-2 Inhibitors (SGLT-2i) | Sodium-Glucose Cotransporter-2 Inhibitors OR SGLT-2i OR canagliflozin OR dapagliflozin OR empagliflozin |
| Bone Fracture | Bone Fracture |

1. Writing the search strategy:

(dipeptidyl peptidase-4 inhibitors OR DPP-4i OR sitagliptin OR vildagliptin OR saxagliptin OR linagliptin OR anagliptin OR teneligliptin OR alogliptin OR trelagliptin OR gemigliptin OR dutogliptin or omarigliptin or exenatide or liraglutide or lixisenatide or albiglutide or dulaglutide or glucagon-like peptide-1 receptor agonists or GLP-1ra OR incretin or sodium-glucose cotransporter-2 inhibitors OR SGLT-2i OR canagliflozin OR dapagliflozin OR empagliflozin) AND fracture.

1. Results

By using this search strategy in the different databases, there were 323 results in **PubMed**, 163 results i**n** **Cochrane Library**, 371 trials in **Scopus,** and 371 results in **Web of Science**.

**Last systematic review and meta-analysis** link: <https://www.frontiersin.org/articles/10.3389/fphar.2022.825417/full>

**Recent studies:**

### The risk of major osteoporotic fractures with GLP-1 receptor agonists when compared to DPP-4 inhibitors: A Danish nationwide cohort study (2022)

### SGLT2 inhibitor treatment is not associated with an increased risk of osteoporotic fractures when compared to GLP-1 receptor agonists: A nationwide cohort study (2022)

1. Fracture Risk of Sodium-Glucose Cotransporter-2 Inhibitors in Chronic Kidney Disease (2022)
2. Fracture risks associated with sodium-glucose cotransporter-2 inhibitors in type 2 diabetes patients across eGFR and albuminuria categories: A population-based study in Hong Kong (2023)

**Final decision**

This idea is valid.

**Screenshots**


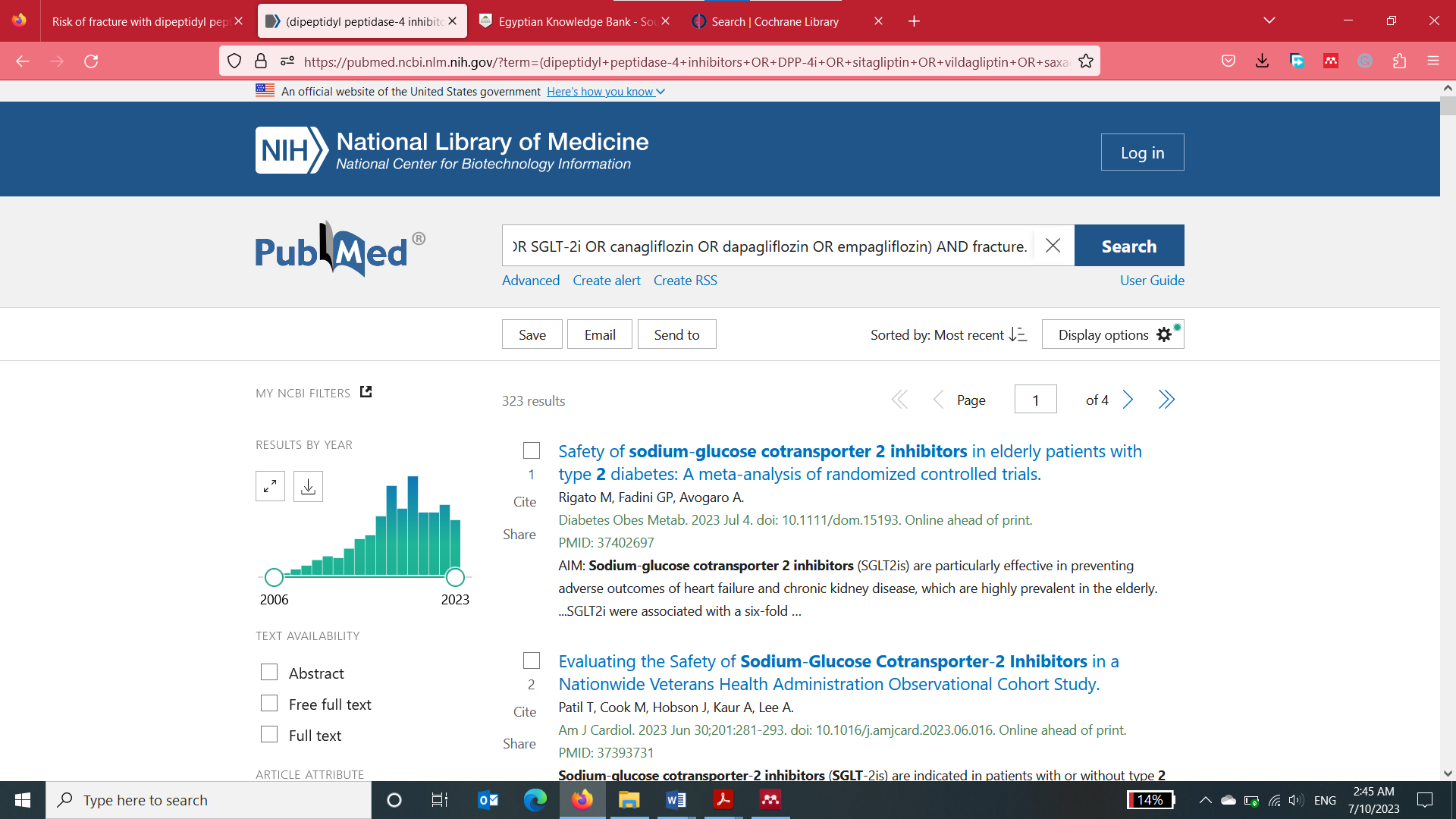


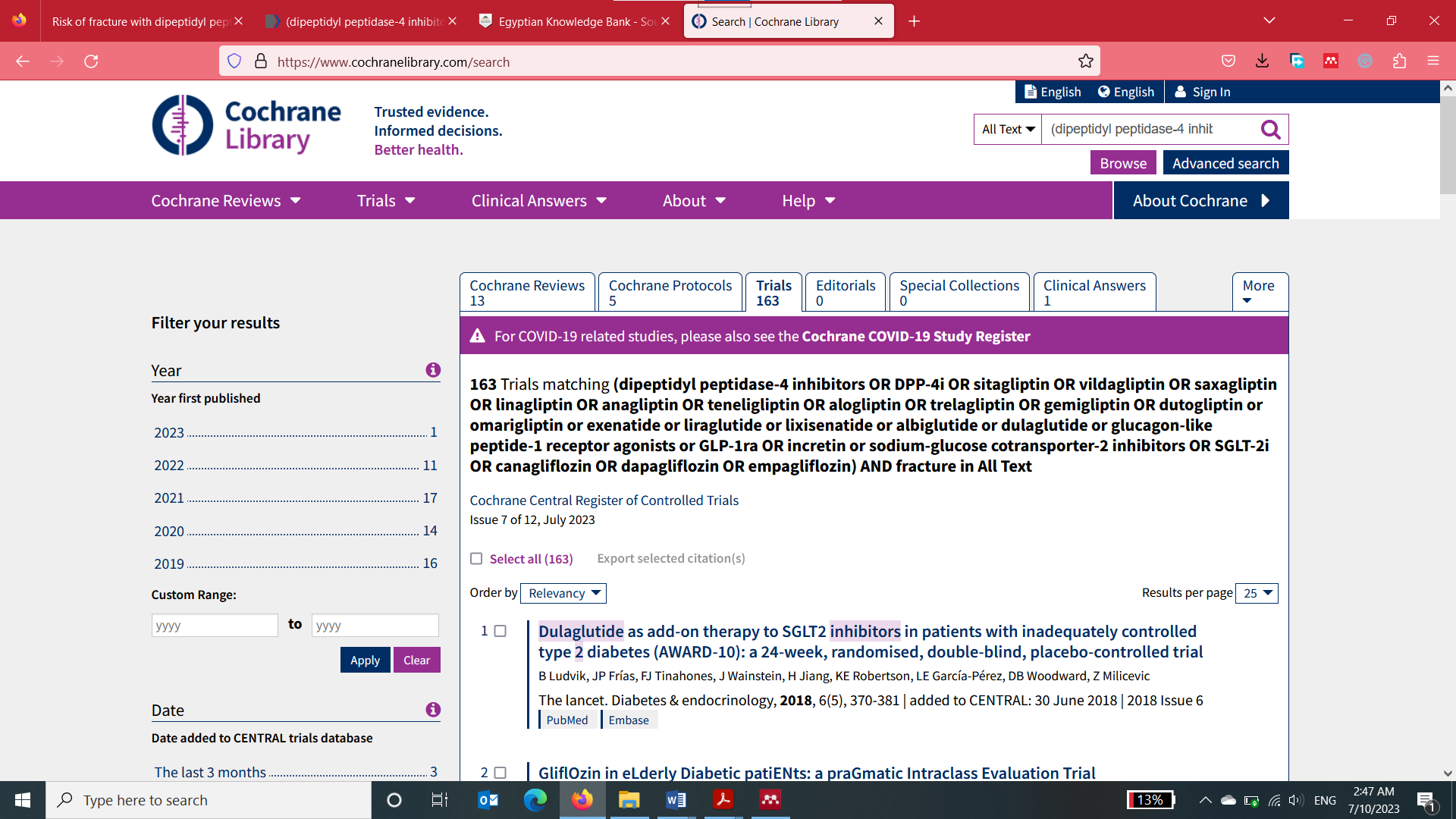


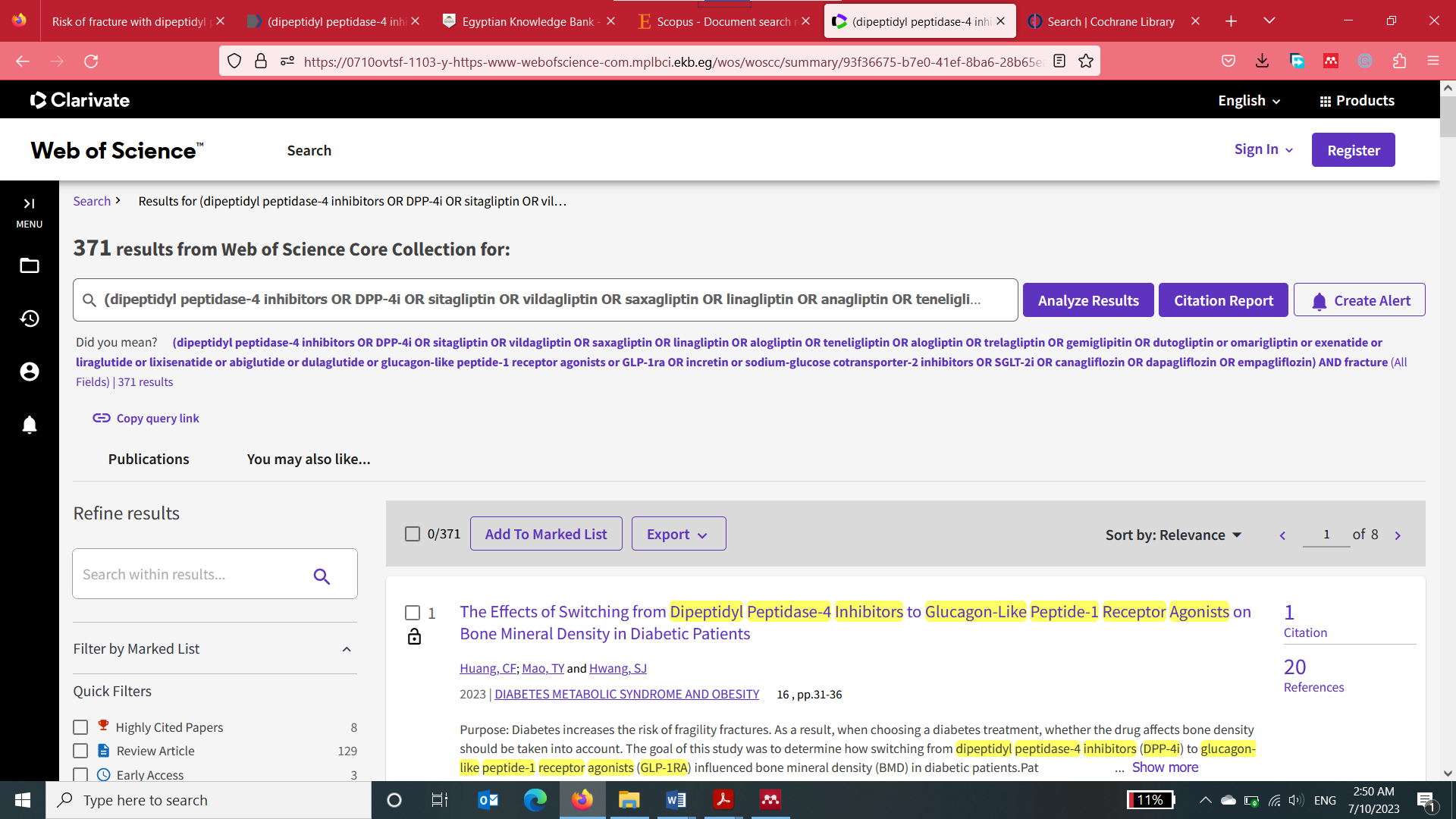


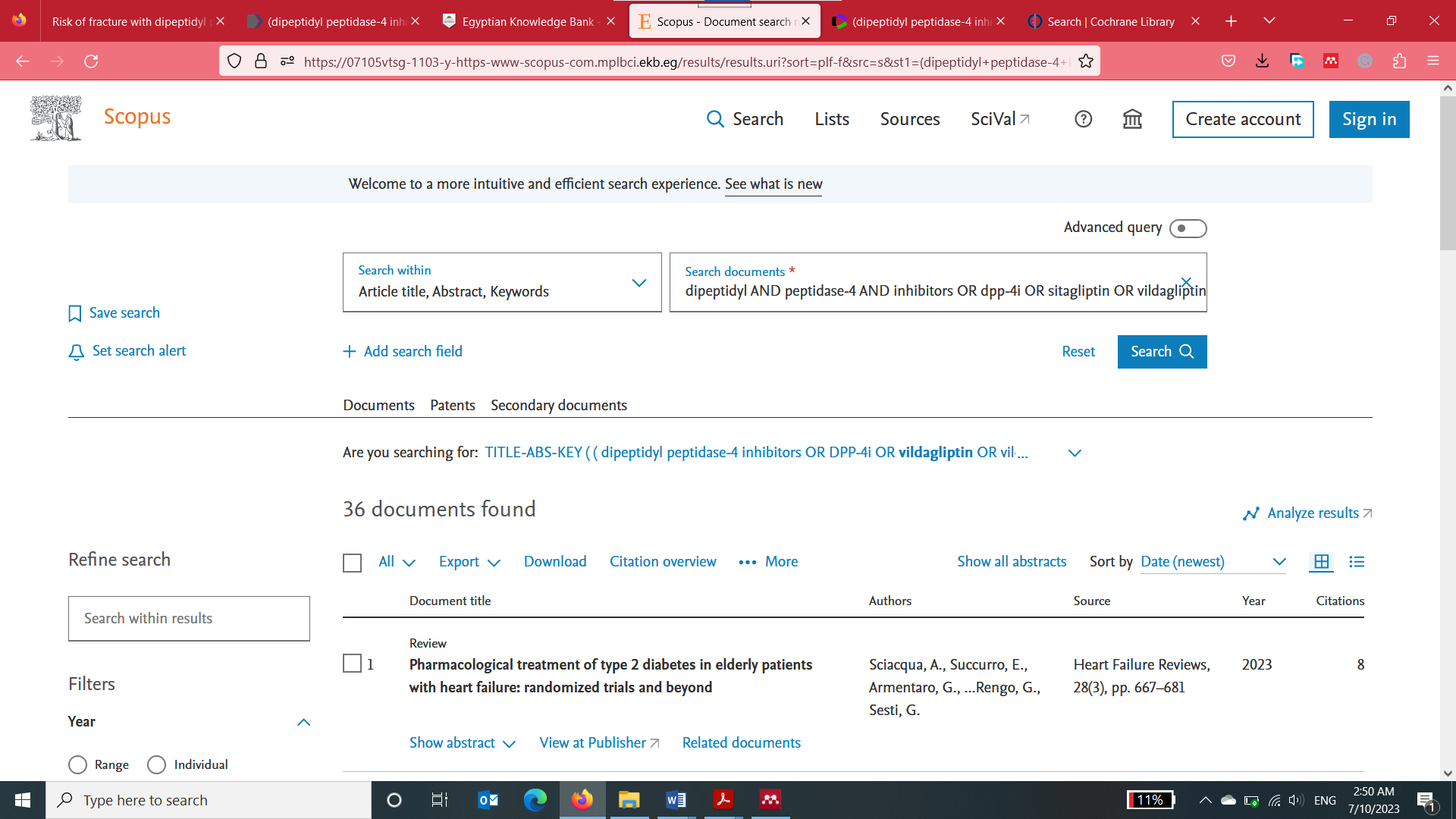

Supplement: Supplementary file 2 [file DataSheet2.docx]
