# Supplementary material for: Risk of bone fracture by using dipeptidyl peptidase-4 inhibitors, glucagon-like peptide-1 receptor agonists, or sodium-glucose cotransporter-2 inhibitors in patients with type 2 diabetes mellitus: a network meta-analysis of population-based cohort studies
Source: Front Endocrinol (Lausanne). 2024 Oct 11;15:1410883. doi: 10.3389/fendo.2024.1410883 (PMC11502341; doi:10.3389/fendo.2024.1410883)
Supplement: Supplementary file 3 [file DataSheet3.docx]

# Newcastle‒Ottawa Quality Assessment Criteria

| **First author, year** | **Selection** | | | | **Comparability** | **Outcome** | | | **Total Score** |
| --- | --- | --- | --- | --- | --- | --- | --- | --- | --- |
|  | **Representativeness of the exposed cohort** | **Selection of the nonexposed cohort** | **Ascertainment of exposure^1^** | **Outcome was not present at start of study^2^** | **Control for 2 important factors^3,4^** | **Assessment of outcome** | **Follow-up long enough** | **Adequacy of follow-up of cohort^5^** |  |
| Al-Mashhadi, 2022 (a) | * | * | * | * | ** | * | * | * | 9 |
| Al-Mashhadi, 2022 (b) | * | * | * | * | ** | * | * | * | 9 |
| Cowan, 2022 | * | * | * | * | * | * | * | * | 8 |
| Driessen, 2015 | * | * | * | * | * | * | * | * | 8 |
| Fralick, 2019 | * | * | * | * | ** | * | * | * | 9 |
| Han, 2020 | * | * | * | * | ** | * | * | * | 9 |
| Lin, 2018 | * | * | * | * | * | * | * | * | 8 |
| Lui, 2023 | * | * | * | * | * | * | * | * | 8 |
| Majumdar, 2016 | * | * | * | * | * | * | * | * | 8 |
| Toulis, 2018 | * | * | * | * | * | * | * | * | 8 |
| Ueda, 2018 | * | * | * | * | * | * | * | * | 8 |
| Zhao, 2021 | * | * | * | * | ** | * | * | * | 9 |
| Zhuo, 2021 | * | * | * | * | ** | * | * | * | 9 |

1 A point was given if the exposure data came from a prescription registry or a medical file.

A point was given if the study was prospective in design.

3 If age adjustments were made, a point was given.

4 If medicines (such as anti-hypertensives, anti-diabetics, etc.) or any other extra considerations were taken into account, a point was given.

5 A point was given if the follow-up was completed with 80% accuracy or more.
